# Anticonvulsive evaluation of THIP in the murine pentylenetetrazole kindling model: lack of anticonvulsive effect of THIP despite functional *δ*‐subunit‐containing GABA_A_ receptors in dentate gyrus granule cells

**DOI:** 10.1002/prp2.322

**Published:** 2017-06-07

**Authors:** Charlotte Simonsen, Kim Boddum, Nadia L. von Schoubye, Alissa Kloppenburg, Kasper Sønderskov, Suzanne L. Hansen, Uffe Kristiansen

**Affiliations:** ^1^ Faculty of Health and Medical Sciences Department of Drug Design and Pharmacology University of Copenhagen Copenhagen Denmark; ^2^ Faculty of Health and Medical Sciences Department of Biomedical Sciences University of Copenhagen Copenhagen Denmark

**Keywords:** Dentate gyrus, GABA, kindling, pentylenetetrazole, THIP, tonic inhibition, *δ*‐containing GABA_A_ receptors

## Abstract

THIP (4,5,6,7‐tetrahydroisoxazolo[5,4‐c]pyridin‐3‐ol) is a GABA_A_ receptor agonist with varying potencies and efficacies at γ‐subunit‐containing receptors. More importantly, THIP acts as a selective superagonist at *δ*‐subunit‐containing receptors (*δ*‐GABA_A_Rs) at clinically relevant concentrations. Evaluation of THIP as a potential anticonvulsant has given contradictory results in different animal models and for this reason, we reevaluated the anticonvulsive properties of THIP in the murine pentylenetetrazole (PTZ) kindling model. As loss of *δ*‐GABA_A_R in the dentate gyrus has been associated with several animal models of epilepsy, we first investigated the presence of functional *δ*‐GABA_A_ receptors. Both immunohistochemistry and Western blot data demonstrated that *δ*‐GABA_A_R expression is not only present in the dentate gyrus, but also the expression level was enhanced in the early phase after PTZ kindling. Whole‐cell patch‐clamp studies in acute hippocampal brain slices revealed that THIP was indeed able to induce a tonic inhibition in dentate gyrus granule cells. However, THIP induced a tonic current of similar magnitude in the PTZ‐kindled mice compared to saline‐treated animals despite the observed upregulation of *δ*‐GABA_A_Rs. Even in the demonstrated presence of functional *δ*‐GABA_A_Rs, THIP (0.5–4 mg/kg) showed no anticonvulsive effect in the PTZ kindling model using a comprehensive in vivo evaluation of the anticonvulsive properties.

**Abbreviations**
aCSF: artificial cerebrospinal fluid
CNS: central nervous system
DGGC: dentate gyrus granule cell
GABA_A_R: GABA receptor subtype A
GABA: γ‐Aminobutyric acid
i.p.: intraperitoneal
IPSC: inhibitory postsynaptic current
I_tonic_: tonic current
OD: optical density
PTZ: Pentylenetetrazole
SR 95531: 6‐Imino‐3‐(4‐methoxyphenyl)‐1(6H)‐pyridazinebutanoic acid
THIP: 4,5,6,7‐tetrahydroisoxazolo[5,4‐c]pyridin‐3‐ol
*δ*‐GABA_A_R: *δ*‐subunit‐containing GABA_A_ receptor

## Introduction

A low‐ambient GABA (γ‐aminobutyric acid) concentration in the extracellular space continuously activates extrasynaptic GABA_A_ receptors, resulting in a persistent inhibitory current causing tonic inhibition (Semyanov et al. 2004; Farrant and Nusser 2005; Glykys and Mody 2007a,b). In many brain regions, this tonic current is primarily mediated by *δ*‐subunit‐containing GABA receptors (*δ*‐GABA_A_Rs), although other extrasynaptic GABA_A_R subtypes may contribute (Brickley et al. 2001; Caraiscos et al. 2004; Jia et al. 2005; Drasbek and Jensen 2006; Glykys and Mody 2006, 2007a). In dentate gyrus granule cells (DGGCs), tonic inhibition is mediated largely by *α*_4_ *βδ*‐GABA_A_Rs with a smaller contribution from *α*_5_ *βγ*‐GABA_A_Rs (Glykys et al. 2008; Liang et al. 2008).

GABAergic neurotransmission is the target of a number of known antiepileptic drugs (White et al. 2007; Madsen et al. 2008). However, the potential of extrasynaptic GABA_A_Rs as targets for antiepileptic drugs has not been elucidated in great detail. Neuroactive steroids with a positive modulatory effect on *δ*‐GABA_A_Rs have been shown to possess anticonvulsant properties (Reddy and Rogawski 2002, 2010). Therefore, the potential anticonvulsant properties of activating the *δ*‐GABA_A_Rs are of great interest.

The GABA_A_ agonist THIP activates GABA_A_R subtypes with varying potency and efficacy (Ebert et al. 1994; Stórustovu and Ebert 2006). Importantly, THIP acts as a superagonist at _α1/4/6_ *βδ* receptors because it has a higher maximum open‐channel probability (= receptor activation) than GABA. This is in turn due to increase in duration of the population of longer channel openings and their frequency, resulting in longer burst durations (Brown et al. 2002; Stórustovu and Ebert 2006; Mortensen et al. 2010).

THIP induces tonic inhibition by activation of *δ*‐containing extrasynaptic GABA_A_ receptors (Herd et al., 2009) and has functional *δ*‐GABA_A_R selectivity at recombinant and native GABA_A_ receptors in clinically relevant concentrations (Drasbek and Jensen 2006; Stórustovu and Ebert 2006; Herd et al. 2009). Furthermore, the hypnotic effect of THIP has been shown to rely on activation of extrasynaptic GABA_A_Rs (Boehm et al. 2006; Herd et al. 2009). The anticonvulsive evaluations of THIP have, however, resulted in contradictory outcomes in different animal models of epilepsy (Löscher and Schwark 1985; Hansen et al. 2004; Madsen et al. 2011).

The hippocampus has an especially high seizure vulnerability, with a relatively low innate after‐discharge threshold in the dentate gyrus and CA1 area (McIntyre and Gilby 2008). C‐fos expression studies have shown that the hippocampus is affected at higher seizure severity scores after PTZ kindling (Erdtmann‐Vourliotis et al. 1998; Szyndler et al. 2009). The *δ*‐subunit has been shown to be downregulated in the dentate gyrus in the late chronic phase in several poststatus epilepticus animal models of epilepsy, which changes the pharmacology but does not reduce the magnitude of the tonic inhibition (Schwarzer et al. 1997; Peng et al. 2004; Zhang et al. 2007; Zhan and Nadler 2009; Rajasekaran et al. 2010). Evidence also exists for *δ*‐subunit mRNA downregulation after electrical kindling in the hippocampus, (Nishimura et al. 2005), although contradictory results exist for both the electrical kindling and poststatus epilepticus models (Kamphuis et al. 1995; Brooks‐Kayal et al. 1998). In the PTZ kindling model GABA_A_ receptor subunit plasticity has only been studied for a few subunits at mRNA level (Follesa et al. 1999), with no investigations of the *δ*‐protein expression. Downregulation of functional *δ*‐GABA_A_Rs in kindling models could therefore potentially account for the lack of anticonvulsive effect of THIP seen in kindling models (Löscher and Schwark 1985; Hansen et al. 2004). In this study we therefore investigated the *δ*‐subunit expression and THIP‐induced tonic inhibition in the dentate gyrus after PTZ kindling and reevaluated the anticonvulsive effect of THIP in the murine PTZ kindling model using a more comprehensive assessment of seizure activity. Some of the results have been published in abstract form (Simonsen et al. 2010).

## Materials and Methods

### Animals

Ethical permission for the studies was granted by the Animal Welfare Committee, appointed by the Danish Ministry of Justice, and all animal procedures were carried out in compliance with the EC Directive 86/609/EEC and with the Danish law regulating experiments on animals.

Three to four weeks old male NMRI mice (Taconic, Denmark) weighing approximately 12–18 g upon arrival were used. They were housed in groups of 2–5 mice in plastic home cages kept on a 12‐h light‐dark cycle (lights on at 6 a.m.). Standard pellet food (Altromin 1314, Brogaarden, Denmark) and tap water were available ad libitum. The mice were allowed to acclimate for approximately 7 days before experimentation, so that they were 4 weeks of age before starting kindling. In vivo experiments were carried out during the light phase of the day.

For immunohistochemistry, Western blot, and electrophysiological recordings, mice were allocated to one of two groups: (1) mice receiving saline injections and (2) mice receiving PTZ injections.

### PTZ kindling

PTZ kindling was performed as previously described (Hansen et al. 2009). Mice were injected with saline or PTZ (Sigma‐Aldrich, Denmark) i.p. in a volume of 10 mL/kg, three times a week (Monday, Wednesday, and Friday) for 4 weeks. During the kindling process, the animals were daily examined by educated animal technicians. In case the animals were exhibiting disease, or misthriving, for example, not drinking or feeding or exhibiting signs of pain, the animal were killed by cervical dislocation.

As sensitivity to PTZ is influenced by seasonal factors (Löscher and Fiedler 1996) and plausibly also biological and technical factors, a dose of 43 mg/kg or 45 mg/kg was administrated to ensure at least 85% of the mice were fully kindled. During the 4th week PTZ‐treated mice were observed for behavioral convulsions for 30 min after PTZ injections. The severity of convulsion was scored according to a modified Racine scale: 0 =  no response; 1 = 1–3 myoclonic jerks and/or facial twitching and/or axial waves going through the body; 2 =  more than 3 myoclonic jerks; 3 =  clonic convulsions with forelimb clonus without loss of postural control; 4 =  clonic convulsions with loss of postural control, turning to the side or rearing; and 5 =  clonic convulsion with loss of righting reflex and/or bouncing, two or more clonic convulsions, tonic convulsions, or status epilepticus.

Mice were considered as fully kindled if they developed clonic convulsions after PTZ injections (scored ≥ 3 on the modified Racine scale) 2 of 3 times during the 4th week. For electrophysiological recordings, saline‐treated mice and fully kindled mice were used day 1–4 after the last injection, whereas for immunohistochemistry and Western blot tissue was harvested at day 3–8 and 7–8, respectively. Only fully kindled mice were used for experimentation and all experiments were performed in parallel on pairs of animals: kindled animals paired with littermates treated with saline.

### Immunohistochemistry

Animals were deeply anesthetized with Hypnorm (Veta Pharma, UK) + Dormicum (midazolam, Accord Healthcare, UK) and intracardially perfused with PBS followed by 4% paraformaldehyde/PBS buffer. Subsequently the brain was removed and postfixed in the same fixative overnight at 5°C. The tissue was cryoprotected in 30% glucose solution for at least 24 h at 5°C, submerged in TissueTek (Sakura, NL), and frozen at −25**°**C before 40‐*μ*m‐thick coronal sections were prepared on a cryostat.

Endogenous peroxidase‐like activity was quenched with 0.3% H_2_O_2_ in blocking solution (PBS containing 1.5% goat serum) and antigen retrieval was carried out by incubation at 90°C for 70 min. Sections were blocked for 30 min in blocking solution and incubated with anti‐GABA_A_R *δ* antibody (1:1000, AB9752; Millipore Corporation) in PBS containing 1.5% goat serum for at least 18 h at RT.

After washing with PBS, sections were incubated 30 min with biotinylated secondary antibody (1:2000, Vector laboratories) and hereafter treated with an HRP‐conjugated avidin enzyme complex (ABC elite, Vector laboratories) for 30 min at RT. Sections were then developed with 3,3′‐Diaminobenzidine (Vector laboratories) and subsequently mounted, dehydrated, and coverslipped before analysis by light microscopy.

Tissue from both kindled and saline‐treated littermates was processed in parallel throughout the staining procedure and images were obtained under identical conditions on the same day.

Images were analyzed and color transformed (conversion from a Black/White scale to a Red/Yellow/Blue scale) using ImageJ software (National Institutes of Health, USA). The optical density (O.D.) of selected brain regions was measured as average of a series of sections from each animal; the molecular layer of dentate gyrus (25–35 sections/mouse), the three outer layers of cortex, and the striatum (10–15 sections/mouse). All values were corrected for background by subtracting a secondary antibody control (treated in parallel but without the primary antibody) from the optical density of the dentate gyrus. The data are presented as the averaged optical density values from each mouse (*n *=* *3).

### Western blot

For Western Blotting, mice were anesthetized with isoflurane vapor (Isoba^®^Vet, Schering‐Plough Animal Health, UK), decapitated, and whole hippocampal tissue was isolated and homogenized in a RIPA buffer containing: 50 mmol/L Tris, 150 mmol/L NaCl, 0.1% SDS, 0.5% Sodium Deoxycholate, 0.1% Triton X‐100, and supplemented with protease inhibitors (Complete Mini, Roche, Switzerland). Samples were centrifuged twice at 40,000 g for 5 min at 4°C and the supernatant was collected for analysis. Protein concentrations were determined using a DC Protein Assay (Bio‐Rad) before protein extracts were supplemented with sample buffer (Kem‐En‐Tec, Denmark), 0.035% 2‐mercaptoethanol, and 2 mmol/L dithiothreitol, and incubated 10 min at 70°C.

Hundred microgram protein was loaded onto a 12% TeoCl SDS Cassette gel (Kem‐En‐Tec, Denmark) and separated in a SDS running buffer (RanBlue, Kem‐En‐Tec, Denmark). The protein was transferred to a pure nitrocellulose membrane (Bio‐Rad, USA) in a transfer buffer (12.5 mmol/L Tris, 15% glycine, 1% SDS, and 15% ethanol).

The GABA_A_ receptor *δ*‐subunit was detected by immunoblotting. The membrane was blocked (PBS containing 2% BSA and 0.1% Tween) and probed with anti‐GABA_A_R *δ* antibody (1:5000, AB9752; Millipore Corporation, USA) at 4°C over night. The blots were then incubated with a goat‐anti‐rabbit peroxidase‐conjugated antibody (1:4000, AB6721; Abcam, UK) and immunoreactive proteins were visualized using Pierce's Enhanced Chemiluminescent substrate (Thermo Scientific, USA). Relative density (RD) of each band was obtained using ImageJ software (National Institutes of Health, USA) and background subtracted (*n *=* *5). RD expressed pr 100 *μ*g protein was used for statistical analysis (Student's t‐test). Tissue from kindled and saline‐treated littermates was placed in neighboring lanes and processed simultaneously on shared membranes throughout the blotting procedure.

### Brain slice preparation

Mice were anesthetized with isoflurane vapor (Isoba^®^Vet, Schering‐Plough Animal Health, UK) and decapitated. A block of the brain containing the hippocampi was dissected and quickly placed in ice‐cold dissection medium (in mmol/L: 248 sucrose, 3.25 KCl, 1.25 NaH_2_PO_4_, 26 NaHCO_3_, 0.5 CaCl_2_, 5 MgSO_4_, 10 D glucose, and bubbled with carbogen (95% O_2_/5% CO_2_); pH~7.4, Osmolality ~350 mOsm/kg) and glued onto the Vibratome stage with cyanoacrylate. Coronal slices (350 *μ*m thick) were cut on a Leica Vibratome (VT1200S, Leica, Germany) and divided into two hemispheres. Slices were stored in an artificial cerebrospinal fluid (aCSF) (in mmol/L: 124 NaCl, 3.25 KCl, 1.25 NaH_2_PO_4_, 26 NaHCO_3_, 2 CaCl_2_, 2 MgSO_4_, 10 d glucose, and bubbled with carbogen; pH ~7.4, Osmolality ~300 mOsm/kg) at 37°C for approximately 15 min and then allowed to recover at room temperature for ≥1 h before recordings.

### Whole‐cell patch‐clamp recordings in brain slices

Slices were placed in a submerged recording chamber and perfused at 2–3 mL/min with 30 ± 2°C warm carbogenated aCSF. Cells located in the DGGC layer were visualized with an infrared Dodt gradient contrast system (IR‐DGC; Luigs & Neumann, Germany) on an upright microscope (BX50WI, Olympus; Japan) equipped with a 60 ×  water immersion objective and a CCD camera (CCD‐300ETRC; DAGE‐MTI, Michigan City, IN).

GABA_A_ receptor‐mediated currents were pharmacologically isolated by adding the nonselective ionotropic glutamate antagonist kynurenic acid (3 mmol/L; Sigma‐Aldrich, Denmark) (Erhardt et al. 2009) to the aCSF and slices were allowed at least 10 min in presence of the antagonist before recordings were started. Patch pipettes were pulled from thin‐walled borosilicate glass (outer diameter = 1.5 mm, World Precision Instruments, Sarasota, FL) on a vertical puller (Model PP‐830, Narishige, Japan). Pipette resistances were 3‐6 MΩ when filled with intracellular solution (in mmol/L: 140 CsCl, 4 NaCl, 1 MgCl_2_, 10 HEPES, 0.1 EGTA, 2 MgATP, 0.3 NaGTP; pH~7.35 with CsOH, Osmolality ~280 mOsm/kg). Whole‐cell recordings were conducted in voltage‐clamp mode at a holding potential of −70 mV (V_h_ = −70 mV) using an EPC 9 amplifier (HEKA, Germany). A stabilization period of at least 7 min was allowed before recordings were started. Series resistance was monitored before and after each experiment, and if series resistance changed > 25% during the recording period or was >20 MΩ, recordings were excluded. Cell capacitance was estimated by the Pulse software (HEKA, Germany), and no significant difference was found in cell size between groups (*P* = 0.21, Student's t‐test). A 65–70% compensation of the series resistance was applied. THIP (1 *μ*mol/L; Gift from Bente Frølund, University of Copenhagen, Denmark) was bath perfused either throughout the recordings or for 5–8 min after baseline recordings (3–6 min) when investigating the effect on sIPSC parameters. Twenty microliter 10 mmol/L stock of the GABA_A_ receptor antagonist SR95531 dissolved in water (Sigma‐Aldrich, Denmark) was then added directly to the bath resulting in a bath concentration > 100 *μ*mol/L.

Currents were low‐pass filtered at 2.9 kHz, digitized at 10 kHz, and stored on a personal computer using the EPC 9 amplifier (HEKA, Germany) and Pulse v8.80 software (HEKA, Germany). Bath application of SR95531 can abolish both GABA_A_ receptor‐mediated phasic and tonic current (Boddum et al. 2014), and was hence used to evaluate these currents. Tonic current was assessed as the outward shift in the holding current. The shift was measured by fitting a Gaussian function to an all‐point histogram of a 10 sec recording segment approximately 20  sec before SR95531 application and to a 10  sec recording segment covering the minimal current recorded after SR95531 application. Before SR95531 application, the current distribution was negatively skewed due to synaptic currents and the Gaussian function was fitted to the unskewed part to exclude spontaneous synaptic events. Total GABA‐mediated tonic current was measured as the size of the SR95531‐induced shift in mean of the Gaussian functions.

sIPSCs were detected in 120 sec recording segments using Mini Analysis Program (Version 6, Synaptosoft Inc) and subsequently inspected visually and corrected when necessary. Inter‐sIPSC interval, 10–90% rise time (RT_10–90_), and peak amplitude were measured and the decay‐time constant (τ_decay_) values were calculated. The sIPSC parameters were obtained from averaged single sIPSCs excluding escaped action potentials and sIPSCs with an inter‐sIPSC interval less than 4–5 x τ_decay_ corresponding to 40 msec for all recordings.

Statistical significance was determined using Student's t‐test (paired or unpaired), one‐way ANOVA, or two‐way ANOVA with repeated measures with *P* < 0.05 as the significance level.

### Whole‐cell patch‐clamp experiments in HEK293 cells

Transient transfections and whole‐cell patch‐clamp recordings were performed as previously described (Madsen et al. 2007) with the following modifications. HEK293 cells were transfected with a combination of human *α*_5_‐pcDNA3.1, *β*_2/3_‐pcDNA3.1, and *γ*_2S_‐pcDNA3.1 (1:1:5 ratio) and cotransfected with plasmid coding for green fluorescent protein using Targefect‐293 as a DNA carrier (Targeting Systems, USA). HEK293 cells were used for patch‐clamp experiments 40–100 h after transfection.

In these experiments we applied agonists for 5–10 sec at 1 min intervals. GABA was applied at a saturating concentration (here 1 mmol/L) eliciting a maximum response in order to monitor the cell's responsiveness and to normalize the other responses. Indeed, every third or fourth application was 1 mmol/L GABA for 5 sec, where the peak response was used to monitor cell responsiveness and update normalization. Sometimes GABA was also applied for 10 sec to see the full time course of the response. It was regularly checked that the receptors recovered from desensitization within the 1 min intervals by applying the same low dose of THIP twice in sequence subsequent to 1 mmol/L GABA.

Occasionally, the potentiation by 1 *μ*mol/L diazepam (Unikem, Denmark) of the GABA‐induced current was determined to ensure the inclusion of the γ_2_‐subunit in the receptor complexes (data not shown).

### In vivo anticonvulsive evaluation of THIP

The potential anticonvulsive effect of THIP was investigated in fully kindled mice in a modified roman square design as previously described (Hansen et al. 2012) with vehicle test conducted twice. THIP (0.5, 1, 2, or 4 mg/kg as the hydrochloride salt) or vehicle (saline 0.9%) was administered subcutaneously (s.c.) 30 min prior to PTZ injection as a THIP exposure study has revealed a maximal CNS concentration within 30–60 min after s.c injection (Cremers and Ebert 2007). All test and vehicle injections had a volume of 10 mL/kg. Mice were observed for 30 min after PTZ injection as described earlier.

The effect of THIP was evaluated on seizure severity as well as incidence of, latency to, and duration of clonic convulsions. For the incidence the standard error was calculated as $\text{S.E.}=\sqrt{\left(\frac{p(1-p)}{n}\right)}$, where p is the proportion of mice with clonic convulsions and *n* is the number of mice (Fowler et al. 1998). Statistical significance compared to vehicle treatment was determined using a z‐test for incidence, χ^2^‐test for seizure severity and one‐way ANOVA for duration and latency with statistical significance level set at *P* < 0.05.

## Results

### Changes in *δ*‐GABA_A_ receptor expression after PTZ kindling

**Figure 1:**
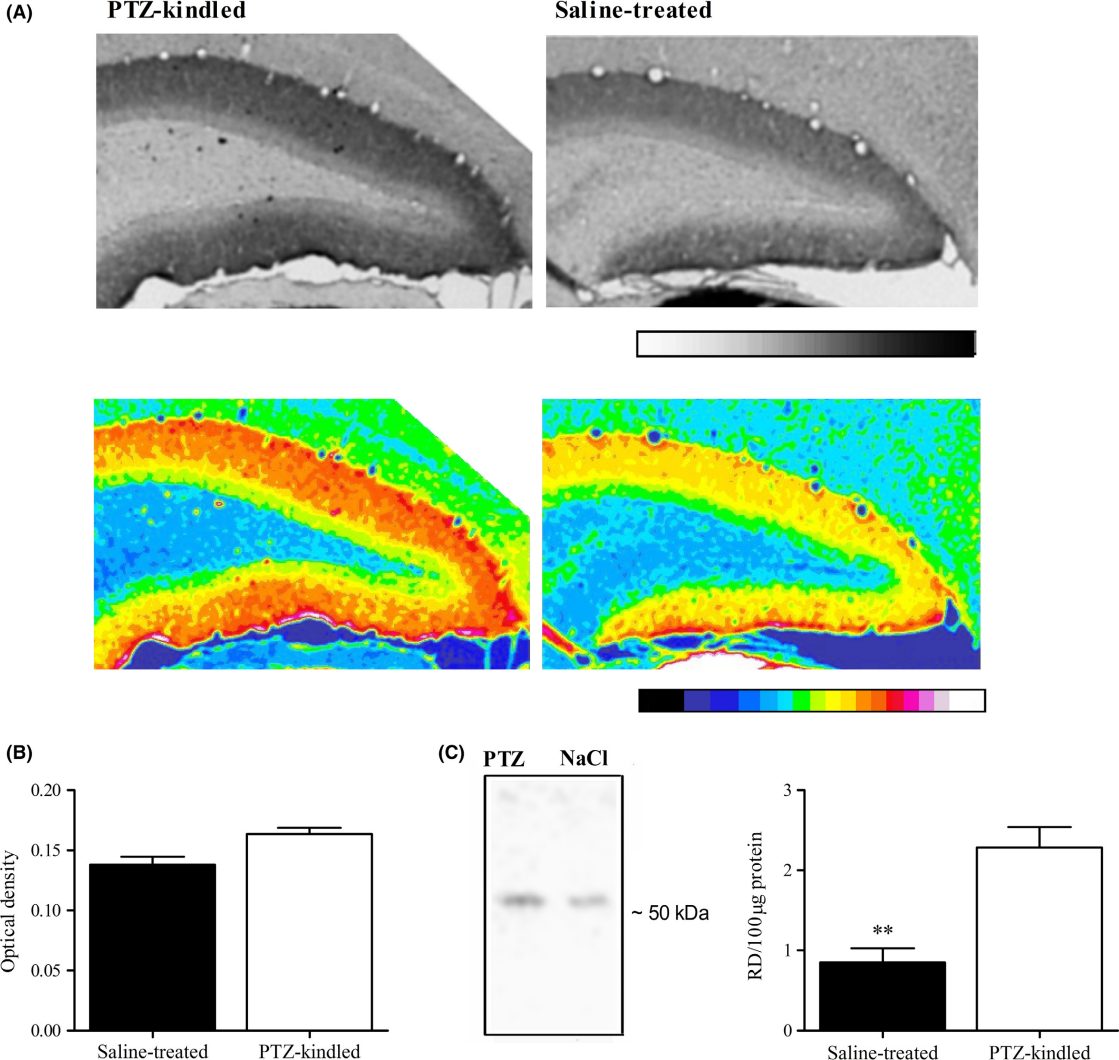
Increased GABA_A_ receptor *δ*‐subunit expression in dentate gyrus after PTZ kindling. (A) Immunohistochemical *δ*‐stainings of the dentate gyrus in gray‐scale and color‐transformed images. The *δ*‐subunit was found to be highly expressed in the molecular layer of the dentate gyrus. The stained brain sections revealed that the density of the *δ*‐subunit is higher in tissue from PTZ‐kindled mice. (B) Bar graph suggesting that the optical density in the dentate gyrus is higher in PTZ‐kindled animals relative to saline treated (25 sections/mouse, *n *=* *3 mice). (C) Representative immunoblots of total protein isolated from hippocampal tissue showing higher *δ*‐subunit expression in PTZ‐kindled animals relative to saline‐treated animals (*left*). *δ*‐levels expressed as the relative density per 100 *μ*g total protein (*right*). The relative density was 61.1 ± 8.4% lower in hippocampal tissue from saline‐treated mice compared to PTZ‐kindled mice (*n *=* *5 animals, **p < 0.01).

In the late chronic phase of poststatus epilepticus animal models of epilepsy, the *δ*‐GABA_A_ receptor subunit has been shown to be downregulated, resulting in changed pharmacology, but not reduced magnitude of the tonic inhibition in DGGCs (Schwarzer et al. 1997; Peng et al. 2004; Zhang et al. 2007; Zhan and Nadler 2009; Rajasekaran et al. 2010). To ensure that the *δ*‐subunit expression is not lost following PTZ kindling, we performed immunohistochemical analysis of brains isolated from PTZ‐kindled and saline‐treated mice using an antibody shown to be specific for the *δ*‐subunit (Maguire et al. 2009). Gray‐scale and spectrum color‐transformed *δ*‐stainings showed a notable staining of the dentate gyrus molecular layer, consistent with the *δ*‐protein predominately being expressed here (Peng et al. 2002). Furthermore, the stainings revealed that the *δ*‐subunit expression was not only present in the molecular layer but also it was increased after PTZ kindling (Fig. 1A). Semiquantitative analysis suggests that the optical density (average of 25–35 section/mouse, 3 mice) was increased in the molecular layer of PTZ‐kindled animals compared to saline‐treated animals (Fig. 1B). In other brain areas rich in *δ*‐expression, such as the striatum and three outer layers of the cortex (Peng et al. 2002), the *δ*‐levels seemed unaltered suggesting that the alteration in *δ*‐expression after PTZ kindling is brain region specific.

To confirm the increased *δ*‐subunit expression after PTZ kindling, Western blot analysis of isolated hippocampi from saline‐treated and PTZ‐kindled animals was performed. In whole‐tissue lysate, the *δ*‐subunit was recognized as a band with a size of ~51 kDa. The OD per 100 *μ*g total protein was significantly increased for PTZ‐kindled animals (Fig. 1C; *n *=* *5 mice, *P* = 0.0017, Student's t‐test). The relative extent of the increase is different between the Western blot and immunohistochemical analysis, which probably arises due to differences in the methodology and method of quantification. For instance, the two methods both include some, however, different nonlinear steps which consequently results in the final read out not being directly proportional to the amount of *δ*‐subunit. Moreover, unspecific primary antibody binding may introduce an error to the immunohistochemical staining which prevents complete background correction. Despite these differences, the two methods consistently showed an upregulation indicating that PTZ kindling causes increased *δ*‐subunit expression in the hippocampus.

### THIP‐induced tonic inhibition in DGGCs

In contrast to the downregulation of *δ*‐subunit expression in the dentate gyrus in the late chronic phase in poststatus epilepticus animal models of epilepsy (Schwarzer et al. 1997; Peng et al. 2004; Rajasekaran et al. 2010), we observed that the *δ*‐subunit was upregulated in the dentate gyrus following PTZ kindling. For this reason, THIP should be able to induce tonic inhibition in DGGCs. We confirmed this by recording the THIP‐induced tonic inhibitory current in DGGCs from PTZ‐kindled as well as saline‐treated mice. THIP of 1 *μ*mol/L was used, as this concentration is within the estimated brain levels of 0.7–3 *μ*mol/L found 30–60 min after s.c. injections of 2.5–10 mg/kg THIP (free base) (Cremers and Ebert 2007). Moreover, this THIP concentration has been shown in other neuronal cell types to activate extrasynaptic GABA_A_Rs but not synaptic GABA_A_Rs (Drasbek and Jensen 2006; Herd et al. 2009).

**Figure 2:**
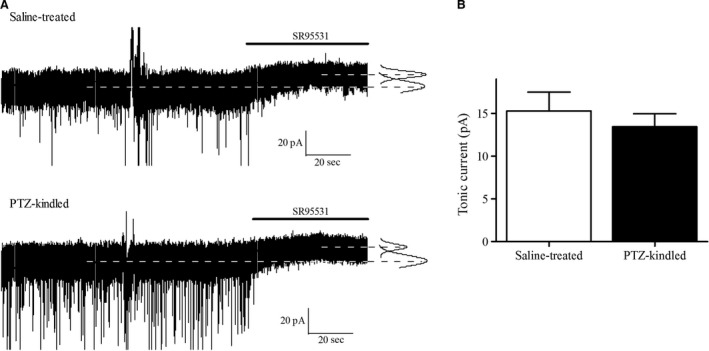
THIP‐induced tonic inhibition in DGGCs from PTZ‐kindled and saline‐treated mice. (A) Representative current traces from DGGC recordings in brain slices from saline‐treated (*upper*) and PTZ‐kindled animals (*lower*) (V_h_ = −70 mV). Some sIPSCs appear truncated. Top lines indicate presence of the GABA_A_ antagonist SR95531. Right, Gaussian fit to the all‐points histogram from 10 s segment with and without the presence of SR95531. (B) The tonic current induced by 1 *μ*mol/L THIP was similar in saline‐treated (*n* = 15 cells) and PTZ‐kindled mice (*n* = 18 cells, *P* > 0.05). Data presented as mean + SEM.

GABAergic inhibition was isolated using the nonselective ionotropic glutamate antagonist kynurenic acid (3 mmol/L). Preliminary data showed that no tonic current was present in DGGCs from either saline‐treated or PTZ‐kindled animals (data not shown), consistent with other studies (Song et al. 2011; Wlodarczyk et al. 2013). This is probably due to a low extracellular GABA concentration arising from washout from the slice preparation (Glykys and Mody 2007b). As a result, when perfusing with 1 *μ*mol/L THIP, the shift in tonic current revealed by application of a saturating concentration of the GABA_A_ receptor antagonist SR95531 equals the THIP‐induced current (Fig. 2A).

The magnitude of the tonic current induced by 1 *μ*mol/L THIP was similar in DGGCs from PTZ‐kindled and saline‐treated animals, with the average total tonic current being 13.4 ± 1.52 pA in PTZ‐kindled mice and 15.3 ± 2.21 pA in saline‐treated mice (Fig. 2B; *n *= 15–18 cells, 6 mice, *P* > 0.05, Student's t‐test). In line with the observed presence of *δ*‐subunit in the dentate gyrus after PTZ kindling, THIP was able to induce a tonic current in DGGCs from both saline‐treated and PTZ‐kindled animals. However, the upregulation of the *δ*‐subunit found in PTZ‐kindled mice did not result in an enhanced THIP‐induced tonic inhibition in DGGCs from PTZ‐kindled mice.

**Figure 3:**
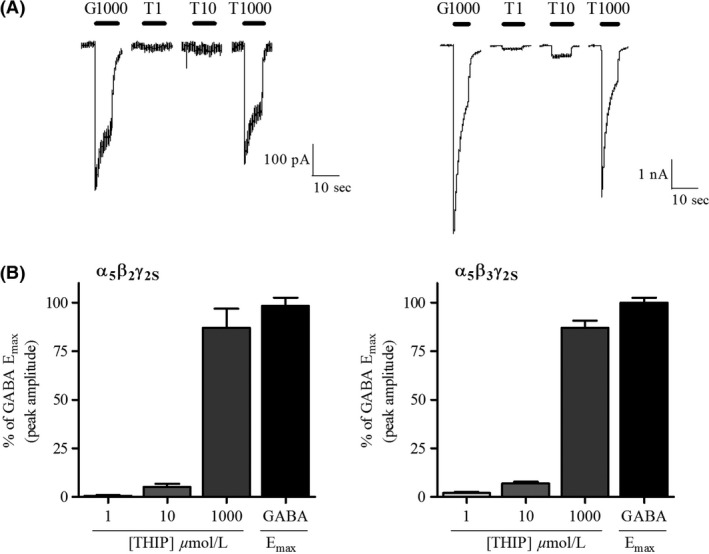
Functional effect of THIP at human recombinant *α*_5_ *β*_2/3_ *γ*_2S_‐GABA_A_ receptors. (A) Representative current traces showing the concentration‐dependent activation of *α*_5_ *β*_2_ *γ*_2S_ (*left*) and *α*_5_ *β*_3_ *γ*_2S_ (*right*) GABA_A_Rs subtypes with different THIP concentrations (T; 1, 10, 1000 *μ*mol/L) compared to 1000 *μ*mol/L GABA (G). (B) The effect of THIP at *α*_5_ *β*_2_ *γ*_2S_ (*n* = 5–6 cells) and *α*_5_ *β*_3_ *γ*_2S_ (*n* = 5–7 cells) GABA_A_R subtypes normalized to the maximal effect of GABA estimated from the GABA concentration response curve (not shown). THIP of 1 *μ*mol/L had minimal effect on α_5_‐GABA_A_Rs, whereas 1000 *μ*mol/L induced a membrane current of ~87% of the maximal effect of GABA, being significantly different from GABA E_max_ only in *α*_5_ *β*_3_ *γ*_2S_ subtype (*P* < 0.05, one‐way ANOVA).

The *δ*‐subunit selectivity of THIP can, however, be questioned as 5 *μ*mol/L THIP induced a residual tonic current in DGGCs from *δ*‐knockout mice (Maguire et al. 2005) and 2 *μ*mol/L THIP induced a current in putative *α*_5_ *βγ*‐GABA_A_Rs in CA1 pyramidal cells (Lindquist et al. 2003). We therefore evaluated the GABA_A_R selectivity of THIP in a recombinant system. As it has been shown that GABA‐induced tonic inhibition in DGGCs is predominately mediated by *δ*‐GABA_A_Rs and to a lesser extent by *α*_5_‐GABA_A_Rs (Glykys et al. 2008), the effect of THIP was tested on human recombinant *α*_5_ *β*_3_ *γ*_2_‐ and *α*_5_ *β*_2_ *γ*_2_‐GABA_A_Rs expressed in HEK293 cells. THIP concentration dependently activated α_5_‐GABA_A_Rs expressed in HEK293 cells (Fig.**** 3A). THIP had an effect of 0.5 ± 0.34% (1 *μ*mol/L), 5.1 ± 1.61% (10 *μ*mol/L), and 87.0 ± 9.78% (1000 *μ*mol/L) relative to the maximum effect of GABA in *α*_5_ *β*_2_ *γ*_2_‐GABA_A_Rs and 2.0 ± 0.56% (1 *μ*mol/L), 6.8 ± 1.02% (10 *μ*mol/L), and 87.0 ± 3.81% (1000 *μ*mol/L) relative to the maximum effect of GABA in *α*_5_ *β*_3_ *γ*_2_‐GABA_A_Rs (Fig. 3B).

Given the minimal effect of 1 *μ*mol/L THIP on *α*_5_ *β*_2/3_ *γ*_2_‐GABA_A_Rs in this study and the observation that THIP potency is highest at the *α*_5_ *βγ*‐GABA_A_Rs of all *αβγ*‐GABA_A_Rs (Stórustovu and Ebert 2006), the THIP‐induced tonic inhibition in DGGCs must primarily be *δ*‐GABA_A_R mediated.

### Phasic inhibition and the effect of THIP in DGGCs

In a number of DGGCs, phasic inhibitory postsynaptic currents and the effect of 1 *μ*mol/L THIP were investigated; PTZ kindling was not found to cause a significant change in the kinetics of the phasic postsynaptic currents in DGGCs (Table**** 1; *P* > 0.05, Student's t‐test).

**Table 1:** Spontaneous inhibitory postsynaptic currents (sIPSCs) recorded in DGGCs from saline‐treated or PTZ‐kindled mice

	Saline treated			PTZ kindled		
	Control	1 *μ*mol/L THIP	% of control	Control	1 *μ*mol/L THIP	% of control
Amplitude (pA)	−25.1 ± 1.92	−24.4 ± 1.10	99.8 ± 6.71	−29.1 ± 3.34	−29.8 ± 2.92	103.6 ± 3.85
RT_10‐90_ (ms)	0.490 [0.407;0.526]	0.543 [0.464;0.945]	140.5 ± 25.01	0.517 [0.444;0.662]	0.534 [0.495;0.622]	115.1 ± 13.93
τ_decay_ (ms)	8.25 ± 0.44	8.94 ± 0.50	108.4 ± 2.69^a^	8.91 ± 0.37	9.59 ± 0.55	108.3 ± 6.03
*n* (cells)	8			10		

Data represented as mean ± SEM, except for RT_10‐90_ (ms) represented as median ± quartiles.

a *P* = 0.016 (paired t‐test)

**Figure 4:**
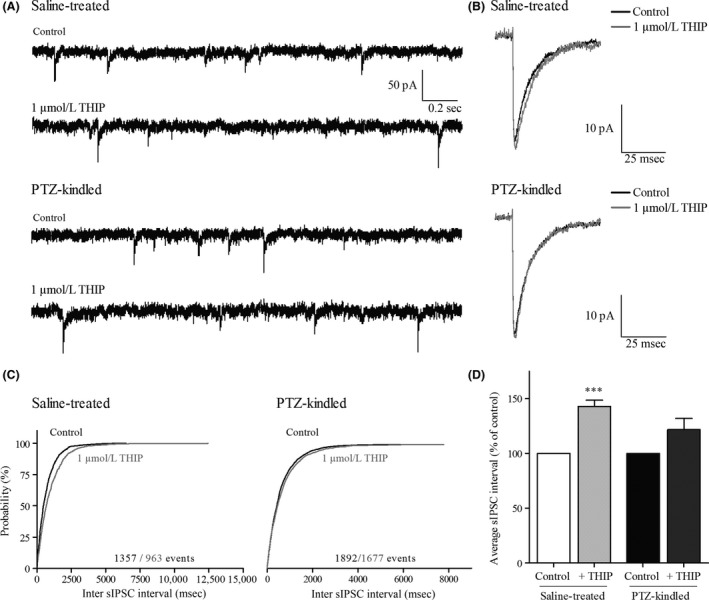
Effect of THIP on phasic currents (sIPSCs) in DGGCs. (A) Representative current traces of 2.5 s of DGGC recordings in control and in presence of 1 *μ*mol/L THIP from the same cell in saline‐treated mice and PTZ‐kindled mice. (B) Superimposed averaged noncontaminated sIPSC from the same cells as in A. (C) Cumulative distribution of inter‐sIPSC intervals in saline‐treated (*n* = 8 cells) and PTZ‐kindled animals (*n* = 10 cells). (D) Effect of 1 *μ*mol/L THIP on the averaged sIPSC interval normalized to the averaged control sIPSC interval obtained from the same cell in saline‐treated (*n* = 8 cells) and PTZ‐kindled mice (*n* = 10 cells). Data presented as mean + SEM. THIP of 1 *μ*mol/L significantly increased the sIPSC interval only in DGGCs from saline‐treated animals (***p < 0.001, paired t‐test).

Application of THIP had no effect on the average sIPSC rise time and amplitude in DGGCs from either saline‐treated or PTZ‐kindled mice (Fig. 4, Table 1; *n *=* *8–10 cells, 4 mice, *P* > 0.05, two‐way ANOVA with repeated measures for amplitude analysis and Kruskal–Wallis one‐way ANOVA on Ranks for rise time analysis). THIP prolonged the IPSC decay in DGGCs (Table**** 1 **)**, however, this effect only reached significance in DGGCs from saline‐treated animals.

THIP induced an increase in the average inter‐sIPSC interval, again only significant in saline‐treated mice (Fig. 4D; *saline treated*: 142.8 ± 5.65% of control, *n *=* *8 cells, *P* < 0.001, paired t‐test; *PTZ kindled*: 121.7 ± 10.22% of control, *n *=* *10 cells, *P* > 0.05, paired t‐test). The cumulative distributions for both treatments are given in Figure 4C (*n* = 8–10 cells). However, no significant effect of PTZ kindling on the sIPSC frequency was observed, neither before nor after THIP application (Basal condition: *saline treated*: 1.41 ± 0.114 Hz and *PTZ kindled*: 1.58 ± 0.146; in presence of THIP: *saline treated*: 1.00 ± 0.096 Hz and *PTZ kindled*: 1.40 ± 0.173 Hz, *P* > 0.05, Student's t‐test, *n* = 8–10 cells).

### Anticonvulsive evaluation of THIP in the PTZ kindling model

Evaluation of THIP as a potential anticonvulsant drug has resulted in contradictory results when evaluated in different animal models of epilepsy. Previously we have shown that THIP had no anticonvulsive effect in the PTZ kindling model when evaluated on seizure incidence (Hansen et al. 2004). However, more recently we found that an anticonvulsive evaluation conducted with several observational parameters offered important additional information about the drug profile that would be lost if only seizure incidence was used as the single evaluation parameter (Hansen et al. 2012). For this reason, we repeated the anticonvulsive evaluation of THIP observing and analyzing several seizure parameters (incidence and duration of convulsions, latency to clonic convulsions, and severity of convulsions) in the PTZ kindling model.

**Figure 5:**
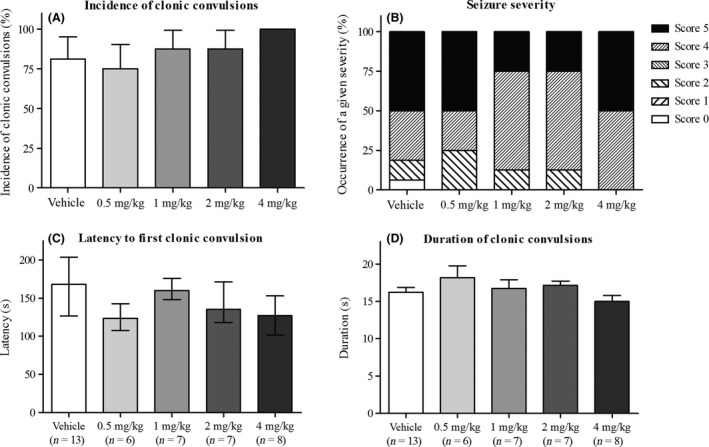
Lack of anticonvulsive effect of THIP (0.5, 1, 2, and 4 mg/kg) in the PTZ kindling model. (A) Incidence of clonic convulsions represented for each treatment as percentage + SE of 8 mice. (B) Seizure severity scored according to the modified Racine scale with Score 0 =  no response; Score 1 = 1–3 myoclonic jerks and/or facial twitching and/or axial waves going through the body; Score 2 =  more than 3 myoclonic jerks; Score 3 =  clonic convulsion with forelimb clonus without loss of postural control; Score 4 =  clonic convulsion with loss of postural control, turning to the side and/or rearing; Score 5 =  clonic convulsion with loss of righting reflex and/or bouncing, two or more clonic convulsions, tonic convulsion or status epilepticus. Data presented for each treatment as percentage of mice achieving the different scores (*n* = 8 mice). (C) Latency to first clonic convulsions presented for each treatment as the median with 25–75% interval. (D) Duration of clonic convulsions represented as mean + SEM. For (C and D) *n* represents the total number of clonic convulsions. In conclusion, THIP showed no anticonvulsive properties on any of the measured parameters.

Despite including the additional parameters in the anticonvulsive evaluation, single administration of THIP in doses up to 4 mg/kg showed no anticonvulsive effect in the PTZ kindling model. THIP had no significant effect on incidence of clonic convulsions (Fig. 5A; *n *= 8 mice, *P* > 0.05, z‐test compared to vehicle treatment) or seizure severity scored according to the modified Racine scale (Fig. 5B; *n *=* *8 mice, *P* > 0.05, χ^2^‐test). For the latency to clonic convulsions, an overall effect of treatments was detected with Kruskal–Wallis one‐way ANOVA on ranks (*P* = 0.036), but no significant pairwise difference between individual treatment groups could be isolated with multiple comparison test (Fig. 5C; *n *=* *6–13 clonic convulsions). THIP did not affect the duration of clonic convulsions (Fig. 5D; *n *=* *6–13 convulsions, *P* > 0.05, one‐way ANOVA). Altogether, none of the parameters used to evaluate seizure activity indicated any anticonvulsant effects of THIP.

## Discussion

In this study we have shown that GABA_A_R *δ*‐subunit expression is not compromised in the dentate gyrus in the early phase (up to 8 days) after PTZ kindling, in fact we found the subunit to be upregulated. THIP was indeed able to induce a tonic inhibition in dentate gyrus granule cells. However, the upregulation of the GABA_A_R *δ*‐subunit was not paralleled by an increase in THIP‐mediated tonic inhibition. THIP induced a tonic current of similar magnitude in DGGCs from saline‐treated as from PTZ‐kindled mice. THIP significantly prolonged the sIPSC decay and reduced the sIPSC frequency in saline‐treated animals; effects that are not significantly different from what was observed in PTZ‐kindled animals. Despite the preserved effect of THIP on tonic inhibition, no anticonvulsive effect of THIP could be detected in the murine PTZ kindling model in a comprehensive in vivo anticonvulsive evaluation.

### Enhanced GABA_A_R *δ*‐subunit expression in dentate gyrus following PTZ kindling

GABA_A_R δ‐subunit plasticity in the dentate gyrus has been observed in the late chronic phase in several poststatus epilepticus animal models of epilepsy employing mice and rats (Schwarzer et al. 1997; Peng et al. 2004; Rajasekaran et al. 2010). In this study we found that GABA_A_R δ‐subunit plasticity also exist in the early phase after PTZ kindling, but in contrast to the downregulation found in poststatus epilepticus models, the GABA_A_R *δ*‐subunit expression appears to be increased in the dentate gyrus in the murine PTZ kindling model.

Immunohistochemistry showed that, in the molecular layer of the dentate gyrus, a prominent *δ*‐staining was present which was enhanced in PTZ‐kindled mice. The GABA_A_R *δ*‐subunit upregulation was confirmed by Western blotting on isolated hippocampal tissue, although the relative extent of the increase was different, likely due to differences in the methodology and method of quantification. Due to the predominant location of the δ‐protein in the molecular layer (shown here and by Peng et al. (2004, 2002)), the upregulation shown by the Western blot analysis probably reflects an altered expression level in the dentate gyrus.

In this study, the GABA_A_R *δ*‐subunit plasticity seemed to be brain‐region specific in the PTZ kindling model as the expression level appeared unaltered in the striatum and cortex. This is in agreement with findings in the pilocarpine‐induced poststatus epilepticus model, where only moderate‐to‐no GABA_A_R *δ*‐subunit plasticity was observed in the striatum, cortex, and thalamus (Peng et al. 2004). Extrasynaptic GABA_A_R plasticity is not only associated with epilepsy models, but has also been found during the estrus cycle (Maguire et al. 2005), pregnancy (Maguire and Mody 2008; Maguire et al. 2009), and schizophrenia (Maldonado‐Avilés et al. 2009; Kjaerby et al. 2014).

### THIP‐induced tonic inhibition in DGGCs

In DGGCs, physiological tonic inhibition is mediated largely by *α*_4_ *βδ*‐GABA_A_Rs with a smaller contribution from *α*_5_ *βγ*‐GABA_A_Rs (Glykys et al. 2008; Liang et al. 2008). The tonic inhibition in DGGCs induced by 1 *μ*mol/L THIP is likely mediated by *α*_4_ *βδ*‐GABA_A_Rs as 1 *μ*mol/L THIP had minimal effect on recombinant *α*_5_ *β*_2/3_ *γ*_2_‐GABA_A_Rs, although a small contribution from *α*_5_‐GABA_A_Rs cannot be excluded.

The GABA_A_R *δ*‐subunit upregulation in the dentate gyrus did not result in enhanced THIP‐induced tonic inhibition in DGGCs in the PTZ kindling model. Instead THIP induced a tonic current of similar magnitude in both saline‐treated and PTZ‐kindled mice, suggesting a preservation rather than an increase in functional *δ*‐GABA_A_Rs after PTZ kindling. The lack of functional *δ*‐GABA_A_R observed in the chronic state of several poststatus epilepticus models (Zhang et al. 2007; Zhan and Nadler 2009; Rajasekaran et al. 2010) must indeed be model specific as a *δ*‐GABA_A_R response to neurosteroids has been found in DGGCs in human temporal lope epilepsy (Scimemi et al. 2006), and functional *δ*‐GABA_A_Rs have been demonstrated in animal models of posttraumatic epilepsy (Mtchedlishvili et al. 2010; Pavlov et al. 2011) and in the PTZ kindling model (as shown here).

The discrepancy between GABA_A_R *δ*‐subunit upregulation and maintained THIP‐induced tonic inhibition in the dentate gyrus could indicate an increased pool of spare *δ*‐GABA_A_R located intracellularly in DGGCs after PTZ kindling. Altered cytoplasmic‐to‐membrane ratio of the *δ*‐protein has previously been shown in pilocarpine‐treated rats, where animals suffering from frequent seizures had a higher level of the GABA_A_R *δ*‐subunit than control animals, when measured in cell lysate obtained from the dentate gyrus (González et al. 2015). However, no significant difference in the level of cell surface localization of GABA_A_R *δ*‐subunits was found between these two groups.

Alternatively, alterations in posttranslational modifications such as the degree of phosphorylation could account for the discrepancy between *δ*‐GABA_A_R expression and function following PTZ kindling. Indeed, diminished THDOC sensitivity of tonic inhibition in layer II pyramidal cells in the piriform cortex after amygdala kindling probably occurred as a consequence of hyperphosphorylation (Kia et al. 2011).

In some studies, PTZ kindling has been shown to increase neurogenesis in the dentate gyrus (Park et al. 2006; Yin et al. 2007; Aniol et al. 2009), whereas several other studies have shown granule cell loss in the dentate gyrus following PTZ kindling (Pohle et al. 1997; Franke and Kittner 2001; Pavlova et al. 2006). If the outcome is an increased number of *δ*‐GABA_A_R‐expressing DGGCs rather than a changed expression of *δ*‐protein in the individual DGGC, the total level of *δ*‐GABA_A_R in the dentate gyrus would be increased, however, the level of tonic inhibition recorded from a single DGGC is left unaltered.

### Effect of THIP on synaptic inhibition in DGGCs

THIP of 1 *μ*mol/L had no effect on the average sIPSC amplitude or rise time. THIP tended to prolong the sIPSC decay time, but this effect only reached significance in DGGCs from saline‐treated animals. This is in line with a small but significant THIP‐induced prolongation of the sIPSC decay time in DGGCs, observed by others (Liang et al. 2006).

THIP also showed a tendency to reduce the sIPSC frequency, but again this was only significant in DGGCs from saline‐treated animals. A THIP‐induced decrease in sIPSC frequency has also been observed in neocortical layer 2/3 pyramidal cells in naive mice consistent with a presynaptic mode of action by decreasing the interneuron firing activity (Drasbek and Jensen 2006; Drasbek et al. 2007). Tonic inhibition of interneurons has been observed in several brain areas (Semyanov et al. 2003; Scimemi et al. 2005, 2006; Glykys et al. 2007) and the lack of a THIP effect on synaptic inhibition of DGGCs in the PTZ kindling model could be a result of either downregulation of *δ*‐GABA_A_Rs on interneurons or reduction in the number of interneurons. Additionally, transient collapse of the chloride gradient and a depolarizing action GABA_A_ receptor activation has been demonstrated in hippocampal interneurons (Song et al. 2011; Ellender et al. 2014), which in this case will cancel or even reverse the effect of THIP.

Both a loss of interneurons and a differential regulation of the *δ*‐subunit expression between principal cells and interneurons have been reported in the pilocarpine‐induced poststatus epilepticus animal model of epilepsy (Kobayashi et al. 2003; Peng et al. 2004), however, with our experimental techniques, it was not possible to resolve such changes.

### Lack of anticonvulsive effect of THIP in the murine PTZ kindling model

In this study we reevaluated the anticonvulsive properties of THIP in the murine PTZ kindling model using a more comprehensive assessment of seizure activity than earlier studies. Despite the presence of functional *δ*‐GABA_A_Rs and the ability of THIP to induce a tonic current in DGGCs, our results indeed confirmed that THIP has no anticonvulsive effect in the murine PTZ kindling model. In doses ranging from 0.5 to 4 mg/kg, THIP showed no change in seizure severity or the occurrence or duration of clonic convulsions. Even though an overall decrease in latency to first convulsion was seen (which may suggest a proconvulsive effect), no significant difference between the different treatment groups could be isolated. Higher doses of THIP were not tested for anticonvulsive effect as doses above 2.5 mg/kg will result in marked sedation (Hansen et al. 2004).

The lack of an anticonvulsive effect of THIP in the PTZ kindling model agrees with previous findings (Hansen et al. 2004), based solely on evaluation of seizure incidence. Lack of anticonvulsive effect of THIP has also been found in female amygdala‐kindled rats (Löscher and Schwark 1985) and in doses up to 41 mg/kg on acute PTZ‐induced seizures (Hansen et al. 2004). In contrast, THIP has shown protective effect against audiogenic seizures (Madsen et al. 2011), and was found to provide better protection against kainic acid‐induced seizures in female mice during diestrus when hippocampal *δ*‐subunit levels are enhanced (Maguire et al. 2005). Even proconvulsive properties have been reported, as THIP was shown to induce absence seizures in rats (Cope et al. 2009). Therefore, the therapeutic potential of extrasynaptic GABA_A_ receptor agonists or modulators as antiepileptic drugs may well be restricted to certain types of epilepsy, perhaps with an associated risk of initiating epileptiform activity elsewhere in the brain.

## Disclosure

None declared.
